# Impacts of the Marine Hatchery Built Environment, Water and Feed on Mucosal Microbiome Colonization Across Ontogeny in Yellowtail Kingfish, *Seriola lalandi*

**DOI:** 10.3389/fmars.2021.676731

**Published:** 2021-05-13

**Authors:** Jeremiah J. Minich, Barbara Nowak, Abigail Elizur, Rob Knight, Stewart Fielder, Eric E. Allen

**Affiliations:** 1 Marine Biology Research Division, Scripps Institution of Oceanography, University of California, San Diego, La Jolla, CA, United States,; 2 Institute for Marine and Antarctic Studies, University of Tasmania, Hobart, TAS, Australia,; 3 Genecology Research Centre, School of Science and Engineering, University of the Sunshine Coast, Sippy Downs, QLD, Australia,; 4 Department of Pediatrics, School of Medicine, University of California, San Diego, La Jolla, CA, United States,; 5 Department of Computer Science and Engineering, University of California, San Diego, La Jolla, CA, United States,; 6 Department of Bioengineering, University of California, San Diego, La Jolla, CA, United States,; 7 Center for Microbiome Innovation, University of California, San Diego, La Jolla, CA, United States,; 8 New South Wales Department of Primary Industries, Port Stephens Fisheries Institute, Nelson Bay, NSW, Australia,; 9 Division of Biological Sciences, University of California, San Diego, La Jolla, CA, United States

**Keywords:** microbiome, built environment, yellowtail kingfish, *Seriola lalandi*, aquaculture, fisheries, ontogeny, mariculture

## Abstract

The fish gut microbiome is impacted by a number of biological and environmental factors including fish feed formulations. Unlike mammals, vertical microbiome transmission is largely absent in fish and thus little is known about how the gut microbiome is initially colonized during hatchery rearing nor the stability throughout growout stages. Here we investigate how various microbial-rich surfaces from the built environment “BE” and feed influence the development of the mucosal microbiome (gill, skin, and digesta) of an economically important marine fish, yellowtail kingfish, *Seriola lalandi*, over time. For the first experiment, we sampled gill and skin microbiomes from 36 fish reared in three tank conditions, and demonstrate that the gill is more influenced by the surrounding environment than the skin. In a second experiment, fish mucous (gill, skin, and digesta), the BE (tank side, water, inlet pipe, airstones, and air diffusers) and feed were sampled from indoor reared fish at three ages (43, 137, and 430 dph; *n* = 12 per age). At 430 dph, 20 additional fish were sampled from an outdoor ocean net pen. A total of 304 samples were processed for 16S rRNA gene sequencing. Gill and skin alpha diversity increased while gut diversity decreased with age. Diversity was much lower in fish from the ocean net pen compared to indoor fish. The gill and skin are most influenced by the BE early in development, with aeration equipment having more impact in later ages, while the gut “allochthonous” microbiome becomes increasingly differentiated from the environment over time. Feed had a relatively low impact on driving microbial communities. Our findings suggest that *S. lalandi* mucosal microbiomes are differentially influenced by the BE with a high turnover and rapid succession occurring in the gill and skin while the gut microbiome is more stable. We demonstrate how individual components of a hatchery system, especially aeration equipment, may contribute directly to microbiome development in a marine fish. In addition, results demonstrate how early life (larval) exposure to biofouling in the rearing environment may influence fish microbiome development which is important for animal health and aquaculture production.

## INTRODUCTION

Aquaculture, which is the farming of aquatic organisms including algae, invertebrates, and vertebrates, has been one of the fastest growing agriculture sectors (8.8% annual growth between 1980 and 2010) for the past 40 years (The State of World Fisheries and Aquaculture, 2020). Demand for seafood has continually grown with global fish production in 2018 at around 179 million metric tons (MMT), of which 82 MMT comes from aquaculture (The State of World Fisheries and Aquaculture, 2020). While 86.5% of total finfish production occurs in inland freshwater systems, with the majority in Asia (The State of World Fisheries and Aquaculture, 2020), marine culture has the highest growth potential with 2% of oceans being suitable for fish farming (Oyinlola et al., 2018). For marine aquaculture growth, Australia, Argentina, India, Mexico, and the United States have the greatest potential based on suitable habitat (Gentry et al., 2017). Freshwater finfish production has primarily been driven by carp, catfish, and tilapia, while marine fish production is dominated by Atlantic salmon which has a freshwater hatchery stage. Despite the recognized opportunities for marine finfish aquaculture production, very few marine fish species have been successful compared to freshwater fish, due in part to the inability to spawn and produce quality fingerlings in captivity. This has led to the common practice of catching wild juveniles and their transfer to captive rearing environments. In recent years, however, certain high value marine species, including the yellowtail kingfish (YTK) *Seriola lalandi*, have been successfully reared in the lab (Welch et al., 2010). The *Seriola* genus, within the family Carangidae, contains several species of yellowtail (Purcell et al., 2015; Oyinlola et al., 2018) that are globally distributed across broad temperature range (Poortenaar et al., 2001). *S. lalandi*, is reared in temperate waters across the Pacific Ocean (Nakada, 2002; Food and Agriculture Organization of the United Nations, 2008; Orellana et al., 2014) in Japan (Nakada, 2002), Australia (Nakada, 2002; Hutson et al., 2007), New Zealand (Orellana et al., 2014; Symonds et al., 2014), Chile (Orellana et al., 2014), and North America (The State of World Fisheries and Aquaculture, 2020).

Fish, unlike mammals, are not thought to inherit their microbiome vertically. Understanding the factors which influence microbiome development in fish is an important first step in mitigating disease and promoting health. One of the primary challenges in marine fish hatcheries is poor survival rate which is often attributed to a combination of disease and nutrition (Sepúlveda et al., 2017). Even in the wild, the survival rate for fish larvae is 44× higher for freshwater fish (5.3%) as compared to marine (0.12%; Houde, 1994). Wild marine fish, particularly temperate coastal pelagics like *Seriola spp*. (Ben-Aderet, 2017), are exposed to wide ranges in environmental variables such as temperature, oxygen, and nutrients both diurnally with vertical migration for feeding and temporally with changing seasons. The mucosal microbiome of coastal pelagics is highly differentiated across body sites, primarily in the gill, skin, digesta, and gut tissue with the microbiome on external sites (gill and skin) most influenced by these changing environmental variables (Minich et al., 2020a). In mammals, both phylogeny and diet influence gut microbiome development (Groussin et al., 2017), whereas fish microbiomes are influenced more by environmental variables including habitat, trophic level, phylogeny, and diet (Sullam et al., 2012; Egerton et al., 2018). Diet also varies widely by development stage particularly in the larval to fry stages (Infante et al., 2000). While mammals have a significant proportion of their gut microbiome colonized or inherited vertically from the mother during birth (Mändar and Mikelsaar, 1996; Dominguez-Bello et al., 2010; Korpela et al., 2018), the initial establishment of the gut microbiome in fish is less understood. Even fewer studies have sought to identify the source colonizers of gill and skin communities.

Microbial colonization throughout development of the fish is a function of both exposure and host selection. At the earliest stage, bacteria which form biofilms on the outside of the egg eventually can colonize both external and internal mucosal sites of freshly hatched larvae upon ingestion of the yolk sac (Hansen and Olafsen, 1999). Marine fish differ from freshwater fish in that they must drink vast quantities of water to maintain osmoregulation, which in turn provides a large source of potential microbes for gut colonization (Hansen and Olafsen, 1999). The first live feeds the larvae consume, which in hatchery settings are often artemia and rotifers, also contribute to the gut microbiome development (Ringø, 1999; Egerton et al., 2018; Wang et al., 2018). In larval YTK, *S. lalandi*, gut microbiome composition and density changes most when transitioning from a live rotifer feed to pellet based feeds around 30 days post hatch (Walburn et al., 2019) with many of the gut microbes having anti-microbial functionality (Ramírez et al., 2019). In a study assessing gut enteritis in farmed *S. lalandi* from seapens, gill, and skin microbiomes correlated with disease state suggesting these communities were either responding to overall health decline or contributing to stress (Legrand et al., 2017). Skin and gut microbiomes of captively reared *S. lalandi* were also influenced by diet and temperature (Horlick et al., 2020). For a freshwater hatchery, the tank side and tank water were shown to significantly influence the skin and gut microbiomes of Atlantic salmon (Minich et al., 2020b). Despite the array of studies evaluating impacts of various husbandry methods on microbiome composition of mucosal sites (gill, skin, and gut), there is a lack of information for how microbiomes on surfaces in the built environment (BE) directly contribute to marine fish.

To evaluate how the collective hatchery microbiome influences the mucosal microbiome of a marine fish, we investigated the economically important YTK *S. lalandi*. This study sought to answer three primary questions: (1) Are body sites differentially influenced by the BE or feed microbiome?, (2) What surfaces within a hatchery environment contribute to the mucosal microbiome of the fish?, and (3) Does the BE and feed microbiome source contribution vary across age and development of the fish? To answer these questions, we sampled the mucosal microbiomes of 92 fish across three broad development stages (fry, pre-stocking juvenile, and mature adult). Specifically, we used 16S rRNA amplicon sequencing of microbial communities from the fish (gill, skin, and digesta “allochthonous”) together with various hatchery surfaces including tank water, tank side, inlet water pipe, air stones, and air diffusers along with feed used in all stages of production. To our knowledge this is the first study to quantify and compare the relationship of the BE microbiome with the fish microbiome across multiple age classes of a marine fish.

## MATERIALS AND METHODS

### Sampling Design

All sampling events occurred in June of 2018 in Port Stephens Australia at the Department of Primary Industries New South Wales. Two broad sampling regimes were carried out (Supplementary Table 1). A total of 92 “YTK” were sampled in Port Stephens, Australia. In the first experiment, gill and skin swabs were sampled from a total of 36 living fish across three different indoor rearing condition tanks (12 fish per tank) along with corresponding BE samples including tank water, the tank side, inlet pipes, and air diffusers. These fish were all siblings and 130 days post hatch “dph.” Fish were reared in either a flow through system “FT,” a traditional moving bed bioreactor “MBBR” Recirculating Aquaculture Systems “RAS,” or a modified BioGill RAS. Fish were reared at a max of 25 kg/m^3^ fed at a maximum of 0.5 kg food/day/m^3^ and reared in 10 m^3^ tanks. Additional details can be found in the white paper (Enabling land-based production of juvenile YTK in NSW). Fish were non-lethally sampled during routine biometric measurements where individuals were weighed and measured. Prior to taking the weight and length, the skin and gill of each fish was swabbed using a cotton swab [Puritan] and placed directly into a 2 ml PowerSoil tube. For these three tank conditions, “BE” samples were taken from the tank water, swab of tank side (biofilm), swab of air diffuser, swab of air stone, and swab of inlet water pipe. For the two RAS tanks, an additional inlet water sample was taken which represents cleaned water (post filtration). Comparisons were made to determine if there was a relationship between the external fish mucosal sites and the BE and if so how that varied across the water filtration or rearing system.

For the second experiment, fish were sampled cross sectionally at different ages including 43 dph (indoor), 137 dph (indoor), and 430 dph (indoor and outdoor). Fish at 430 dph included fish sampled from an ocean net pen along with fish which were transferred from an ocean net pen back to an indoor system. For the age comparison cohort, three body sites were sampled including the gill, skin, and digesta “allochthonous” samples along with corresponding BE samples described in experiment 1. The BE “built environment” samples included tank water, inlet pipe, airstone, air diffuser, and tank side. Specifically 12 fish were similarly non-lethally sampled from three different age classes: 43, 137, and 430 dph from indoor tanks. The 430 dph fish from the indoor tank were initially reared indoor until 245 dph following methods described by Stewart Fielder et al. (2011) and then transferred to ocean netpens where they were grown for 106 days. At 351 dph, they were then transported back to the indoor system where they were held until sampled at 430 dph. An additional 20 fish at 430 dph from the seapen were harvested for another experiment and opportunistically sampled. All fish were measured for length and mass with condition factor calculated. A total of 92 fish were sampled across the two experiments. For the entire experiment, 304 samples were processed for DNA extraction including 19 controls, 45 “BE” samples, 92 gill swabs, 92 skin swabs, and 56 digesta swabs (Supplementary Table 1).

### Microbiome Sample Preparation and Processing

After swabbing the BE and fish mucosal sites, individual swab heads were broken off into a 2 ml PowerSoil tube and then stored at −20°C for 2 weeks until DNA extraction to preserve microbiome integrity (Song et al., 2016). All molecular processing was done according to the standard Earth Microbiome Project protocols (Thompson et al., 2017; earthmicrobiome.org). Batches of samples were extracted in groups of 48 using the Mobio PowerSoil kit (Cat# 12888–50). Lysis in single tubes were used to minimize noise from well-to-well contamination (Minich et al., 2019; Walker, 2019). A serial dilution (titration) of a positive control, *Escherichia coli* isolate (*n* = 12), along with negative control blanks (*n* = 7) were included to estimate the limit of detection of the assay (Minich et al., 2018b). By using the Katharoseq method, we empirically calculated the read count used to exclude samples (Minich et al., 2018b). For library preparation, DNA samples of equal volume (0.2 μl) were processed using the EMP 16S rRNA 515F (Parada)/806R (Apprill) primers (Caporaso et al., 2011; Apprill et al., 2015; Parada et al., 2016; Walters et al., 2016) with 12 bp golay barcodes at a miniaturized PCR reaction volume of 5 μl reactions in triplicate (Minich et al., 2018a). After PCR, equal volumes of each library (2 μl) were pooled and processed through the MinElute PCR purification kit (Qiagen Cat# 28004) followed by a 1× Ampure cleanup. The final library was sequenced using a MiSeq 2 × 250 bp kit (Caporaso et al., 2012).

### Microbiome Analysis

Sequences were uploaded, demultiplexed, and processed in Qiita (Gonzalez et al., 2018), using the Qiime2 commands (Bolyen et al., 2019; Estaki et al., 2020). Specifically, sequences from the first read were trimmed to 150 bp following the EMP protocol, and processed through the deblur pipeline and SEPP (Janssen et al., 2018) to generate Amplicon Sequence Variants “ASVs” (Amir et al., 2017). ASVs were rarified to 5,000 reads per sample. General Alpha and Beta diversity measures (Whittaker et al., 2001; Reese and Dunn, 2018) were generated in Qiita. Microbial Alpha diversity comparisons (Reese and Dunn, 2018) were calculated for richness, Shannon diversity (Shannon, 1948), and Faith’s Phylogenetic Diversity (Faith, 1992). For statistical analysis, grouped comparisons (>2 groups) were compared using Kruskal–Wallis test (Kruskal and Allen Wallis, 1952) with Benjamini Hochberg FDR 0.05 (Benjamini and Hochberg, 1995). To compare the age of fish with alpha diversity metrics, both linear regression and Spearman correlation (Spearman, 1904) were used using PRISM 9.0 (La Jolla, CA, United States). Beta diversity measures were calculated using both Unweighted UniFrac and Weighted normalized UniFrac (Hamady et al., 2010; Lozupone et al., 2011). Categorical group comparisons of beta diversity were calculated using PERMANOVA tests (Anderson, 2001, 2017). Lastly, to quantify the effects or sources of microbes from the BE onto the fish mucus, we applied the microbial source tracking software SourceTracker2 (version 2.0.1; Knights et al., 2011). Prior to SourceTracker2 analysis, ASVs which had less than 100 total counts across the dataset were removed to reduce sparsity and improve performance of the microbial source tracking.

## RESULTS

### Microbiome Sequence Data

Both negative and positive controls were used to determine the overall limit of detection to exclude or include samples. Serial dilutions of positive controls indicated a sample exclusion criterion of 2,406 reads (Supplementary Figure 1). To be conservative, we choose to rarefy at 5,000 reads which yielded a total of 246 samples (out of the original 304 samples) and 17,348 unique ASVs. After removing controls, a total of 236 samples were retained resulting in 17,161 ASVs. This includes two primary datasets: the tank rearing comparison of fish at 130 dph (gill, skin, and BE × three tank types) and the age comparison of fish sampled at 43, 137, and 430 dph (gill, skin, digesta, and BE). Overall, sample success was very high (Supplementary Table 1).

### Impact of Rearing System (FT vs RAS) on Fish Mucosal Microbiome (at 130 dph)

**FIGURE 1 |:**
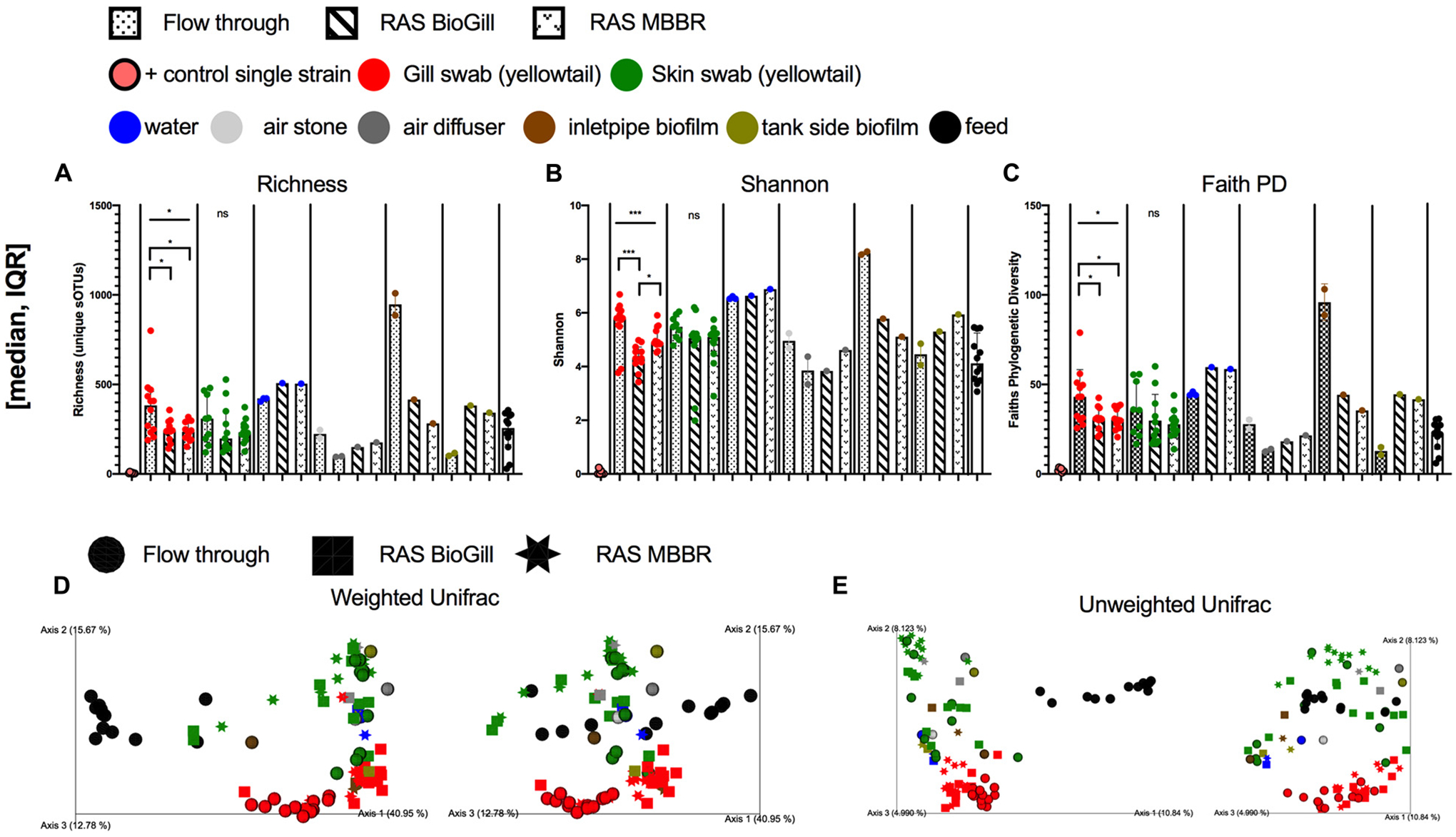
Microbial diversity of the hatchery built environment along with fish gill and skin mucus at 130 days post hatch across three rearing tanks (flow through, RAS BioGill, and RAS MBBR). Alpha diversity as measured by **(A)** richness, **(B)** Shannon, and **(C)** Faith’s phylogenetic diversity. Gill and skin (group comparison calculated with Kruskal–Wallis test, Benjamini Hochberg FDR 0.05). Beta diversity calculated using **(D)** Weighted normalized UniFrac and **(E)** Unweighted UniFrac distance. (*P < 0.05, **P < 0.01, ***P < 0.001, and ****P < 0.0001).

To first assess how the rearing condition influences the microbiome of the BE and external mucosal sites of the fish (gill and skin), 12 YTK (130 dph) fish and various tank controls were sampled from three unique rearing systems. Microbial diversity in the gill varied across tank systems for richness (Figure 1A: *P* = 0.0376, KW = 6.563), Shannon (Figure 1B: *P* = 0.0008, KW = 14.26), and Faith’s Phylogenetic diversity (Figure 1C: *P* = 0.0273, KW = 7.199) with FT grown fish having slightly higher microbial diversity compared to RAS reared. Skin samples did not differ in microbial diversity based on rearing type. In the BE, water generally was highest in microbial diversity, while both air stones and air diffusers had the lowest diversity across all sample types. When comparing the water communities of the FT and RAS tanks, the richness and phylogenetic diversity trended higher in RAS (Figures 1A–C). Interestingly, the inlet pipe biofilms were highly variable across the FT and RAS systems with the FT tank having a very high microbial diversity compared to RAS systems. The tank side biofilms were generally higher in microbial diversity in the RAS tanks as compared to the FT tank. When comparing beta diversity, the largest compositional differences were due to the feed vs all other sample types, with most feed pellet communities highly differentiated from the BE and fish mucus with the exception of live rotifer feeds. Many chloroplasts ASVs were present in the pellet feeds, likely from plant ingredients, which likely drove this separation. Upon chloroplast removal, read counts for feed samples drop to levels which would largely exclude them from analysis thus suggesting that feed samples have very low proportions of microbes. The second largest driver in microbial community composition was the fish body sites for both Weighted and Unweighted UniFrac (Figures 1D,E). For individual body sites, the tank systems also had a moderate impact with gill samples being more differentiated across tank systems (Table 1). Specifically, for gill samples, the tank rearing system had an impact on the microbial community for both Unweighted Unifrac distance (Table 1, PERMANOVA, *P* = 0.001, and *F* = 2.72) and Weighted normalized Unifrac distances (Table 1, PERMANOVA, *P* = 0.001, and *F* = 11.01). Pairwise comparisons of Unweighted Unifrac distances revealed that gill microbiomes of RAS reared fish were also differentiated but in general less differentiated as compared to the FT reared fish (Figure 1E and Table 1). Pairwise comparisons of Weighted normalized Unifrac distances revealed the same pattern, with fish reared in different RAS systems having a differentiated community but more even more differentiated when compared to fish reared in FT systems (Figure 1D and Table 1). Skin microbial communities were only influenced by the rearing method when comparing Unweighted Unifrac (Table 1) but not with Weighted normalized Unifrac. When comparing YTK from the same age and genetic cohort reared in three different conditions, gill microbial communities were more influenced by the environmental conditions than the skin, while microbial communities of the BE were highly variable across tank systems.

### Impact of Age on Fish Mucosal Microbiome

**FIGURE 2 |:**
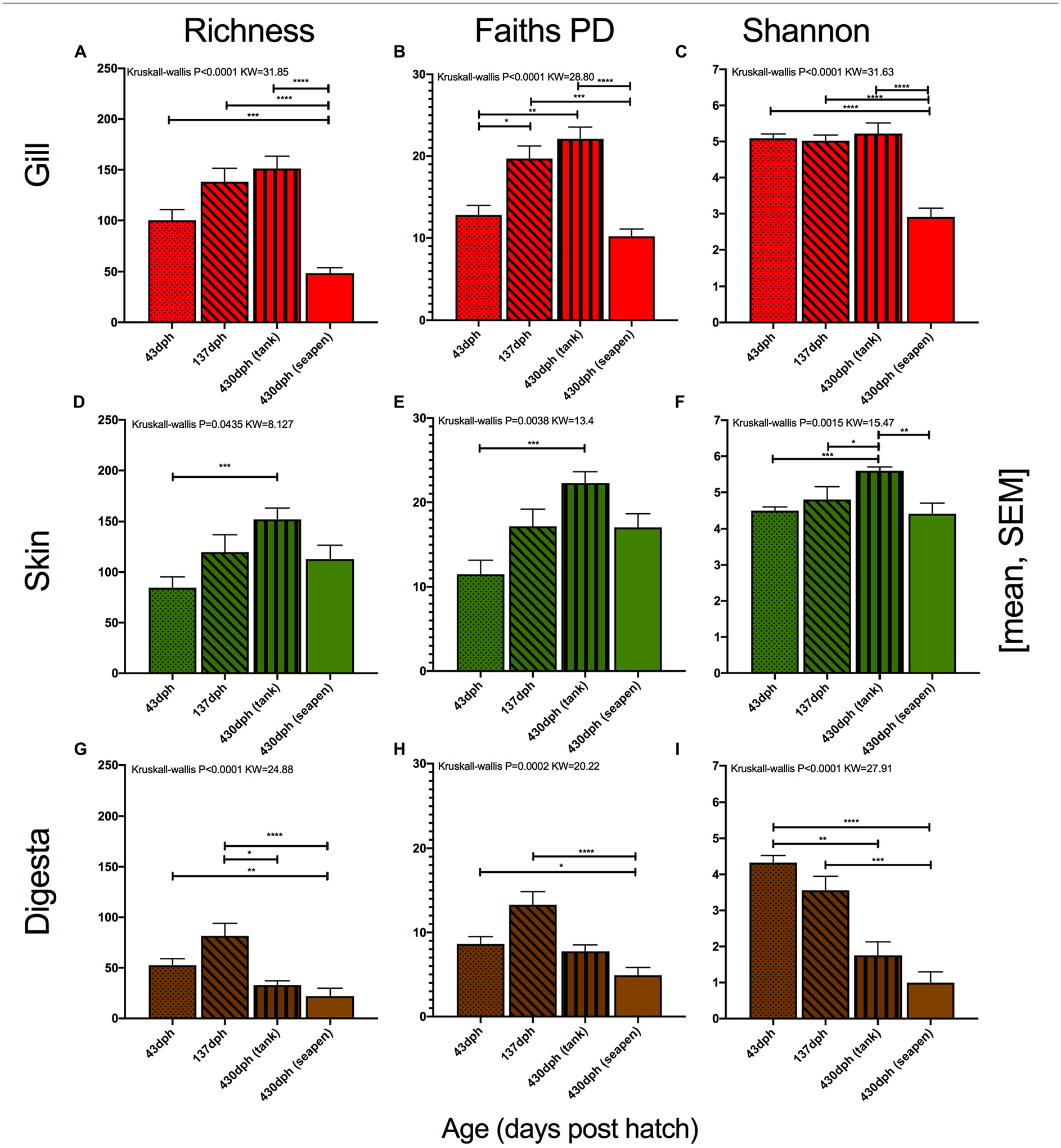
Alpha diversity measures: richness, Faith’s Phylogenetic diversity, and Shannon diversity grouped per body site (red = gill, green = skin, and brown = digesta). Each body site assessed for diversity differences across age (Kruskal–Wallis, Benjamini-Hochberg FDR 0.05). Gill microbial diversity: **(A)** richness, **(B)** Faiths PD, and **(C)** Shannon; Skin microbial diversity: **(D)** richness, **(E)** Faiths PD, and **(F)** Shannon; and Digesta microbial diversity: **(G)** richness, **(H)** Faiths PD, and **(I)** Shannon. (*P < 0.05, **P < 0.01, ***P < 0.001, and ****P < 0.0001).

After quantifying the variation which existed across tank systems at a single age of fish, we next wanted to evaluate the extent by which mucosal microbiomes (gill, skin, and gut) varied with fish age. Specifically, we sought to investigate factors governing the randomness vs. deterministic mechanisms for microbial colonization in marine fish over time. Fish were sampled at three age points including 43, 137, and 430 dph. At 430 dph, fish were either collected from an offshore sea pen (*n* = 20) or from the indoor environment. The indoor fish at 430 dph had been in the sea pen but were transferred back to the indoor environment to be used as broodstock (*n* = 12). These fish were in the indoor tanks for 79 days before sampling. Fish from 43 to 137 dph were always reared in indoor systems. At each body site: gill (Figures 2A–C), skin (Figures 2D–F), and digesta (Figures 2G–I), microbial diversity was compared across fish ages. Additionally, fish from 430 dph were separated by either indoor or ocean net pen. When comparing richness measures, all three body sites were influenced by age with the gill (*P* < 0.0001, KW = 31.85, Figure 2A) being most influenced followed by digesta (*P* < 0.0001, KW = 24.88, Figure 2G) and then skin (*P* = 0.0435, KW = 8.127, Figure 2D). A similar pattern was observed for Faith’s PD, which takes into account microbial phylogenetic diversity with all three body sites being influenced by age. The gill was most influenced (*P* < 0.0001, KW = 28.8, Figure 2B) followed by digesta (*P* = 0.0002, KW = 20.22, Figure 2H) and lastly skin (*P* = 0.0038, KW = 13.4, Figure 2E). Shannon diversity had the same pattern with gill (*P* < 0.0001, KW = 31.63, Figure 2C), digesta (*P* < 0.0001, KW = 27.91, Figure 2I), and skin (*P* = 0.0015, KW = 15.47, Figure 2F) all being influenced by fish age in the same order of impact. When comparing only samples at 430 dph, gill diversity (richness, Faith’s PD, and Shannon evenness) was larger for fish which were transferred from the ocean net pen back into the indoor environment as compared to ocean net pen reared fish. This effect was also seen in the skin, but to a much smaller degree.

**FIGURE 3 |:**
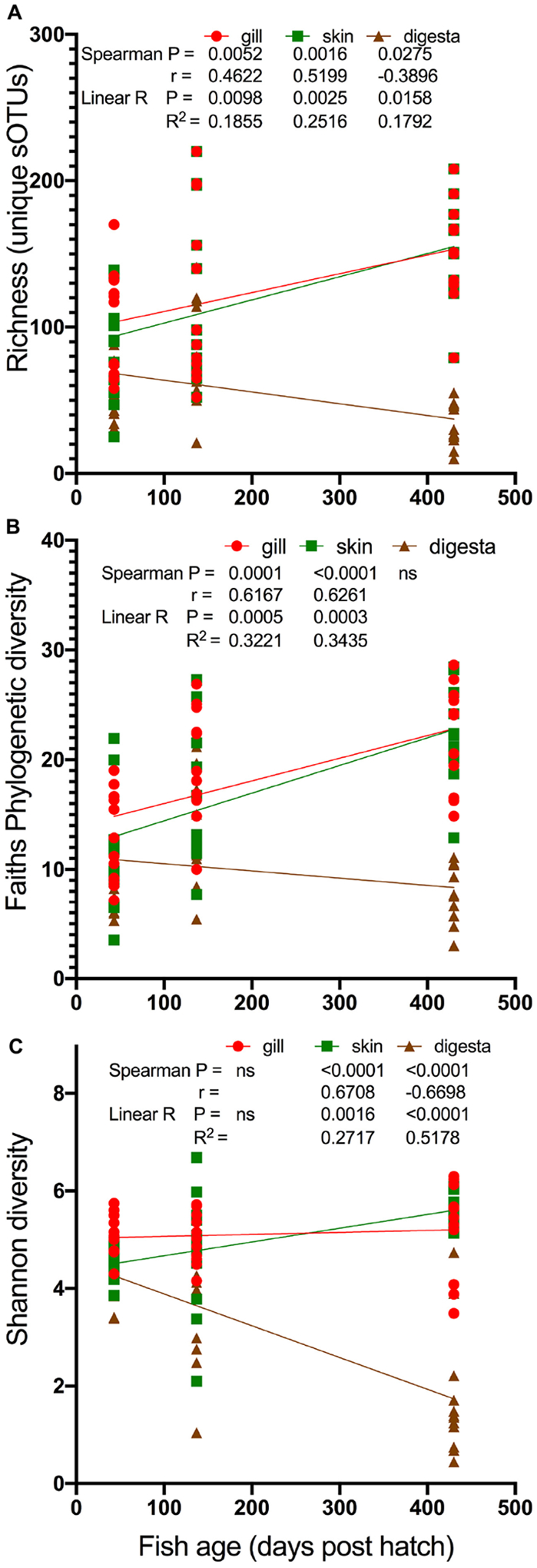
Modeling of changes in alpha diversity: **(A)** richness, **(B)** Faiths PD, and **(C)** Shannon diversity over the age of the fish. Only fish reared in indoor systems included (430 dph seapen fish excluded). Statistical comparisons of both Spearman correlation and linear model (linear regression) calculated with results depicted on the legends.

To model age and microbial diversity across the body sites, we performed a regression and Spearman correlation for each diversity measure. For this analysis, we excluded ocean net pen reared fish from 430 dph to compare only indoor fish (Figure 3). For richness, both gill and skin samples were positively associated with fish age while digesta samples were negatively associated with fish age (Figure 3A). For Faith’s PD, both gill and skin again were positively associated with fish age (Figure 3B). Lastly for Shannon diversity, skin was positively associated with fish age while digesta was negatively associated with fish age (Figure 3C). These cumulative results suggest a general mechanism for alpha diversity changes in the marine fish YTK, *S. lalandi*, whereby alpha diversity may continue to increase over time in the gill and skin surfaces while digesta samples start highly diverse but then adapt or reduce in complexity over time.

### Microbial Compositional Drivers Across Age and Rearing Condition

Next we wanted to understand how the composition of microbial diversity changed over time (age) and to also determine if there was evidence for succession. To determine if age was associated with microbial niche differentiation across body sites, we compared the fish body site microbiome independently at each of the four ages or conditions including 43 dph (Supplementary Figures 3a,b), 137 dph (Supplementary Figures 3c,d), 430 dph “indoor tank” (Supplementary Figures 3e,f), and 430 dph “seapen” (Supplementary Figures 3g,h). Body sites at each age group, even as early as 43 dph, had unique microbial communities measured using Unweighted and Weighted normalized Unifrac distance metrics (Supplementary Table 2a). For Weighted normalized Unifrac, based on the *F*-statistic, body site microbial communities were most differentiated at 430 dph, especially in the open sea pens. This result suggests that body site microbial communities continue to differentiate throughout the lifetime of the fish.

We then sought to answer the question if certain body sites are more influenced by age. To do this, we compared microbiome differences of age and tank type within each body site independently (Supplementary Figures 3i–n and Supplementary Table 2b). For both Unweighted and Weighted normalized Unifrac distance comparisons, the gill microbiome samples were more differentiated across ages as compared to the skin and digesta (*F*-statistic). Furthermore, when observing the gill samples, the 430 dph fish reared in the indoor tank and ocean net pen were divergent on the PCoA (Supplementary Figures 3i,j). In addition, fish at 43 dph were also differentiated.

**FIGURE 4 |:**
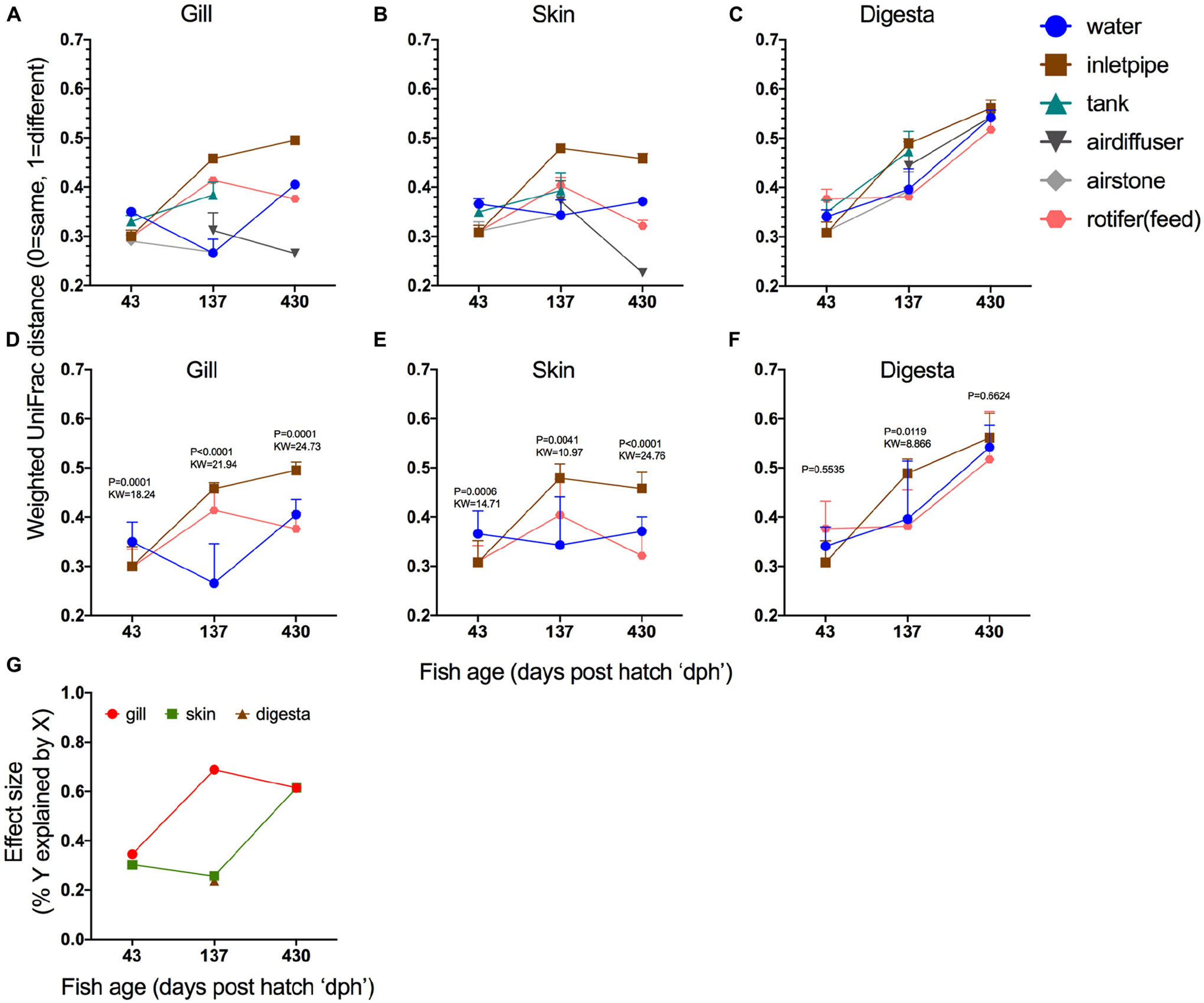
Niche differentiation within body sites over time. Beta diversity distances (Weighted normalized UniFrac) of **(A)** gill, **(B)** skin, and **(C)** digesta samples compared to six different hatchery built environment putative microbial sources [water, inlet pipe, tank side, air diffuser, airstone, and first feed (rotifers)]. Statistical comparison of microbiome differentiation across three BE comparisons (water, inlet pipe, and first feed) over time and calculated independently across three body sites: **(D)** gill, **(E)** skin, and **(F)** digesta (Statistical test: Kruskal–Wallis, *P* value and KW test statistic reported in figure panel. **(G)** Results from the Kruskal–Wallis test for (d,e,f) depicted as effect size to demonstrate the rate of microbial community niche differentiation.

Next, we evaluated if overall fish mucosal microbiome similarity to the BE changed with age and if it did, which BE or water sample types were most influential (e.g., potential source reservoirs for fish microbiome colonization). For indoor reared fish at 43, 137, and 430 dph, we compared the microbiome of the gill, skin, and gut to various hatchery components including tank side, water from the tank, the inlet pipe into the tank, air stones, air diffusers, and feed. For feed, we evaluated 12 different feed types that were used throughout the production schedule ranging from days 1–12 (first feed) until harvest. The first feed type (live rotifers) consistently had a more similar microbial community to the gill, skin, and digesta samples across the different ages (Supplementary Figure 4) thus we used these samples (unenriched and enriched rotifers) for the feed comparison in the broader BE comparison. When including all possible BE sample types, a noticeable trend emerged where at the earliest age (43 dph), the microbial communities across all body sites were generally more similar to the BE (Figures 4A–C). Whereas at later ages, the microbiome of the gill and skin communities generally become more dissimilar from the inlet pipe and feeds, but became more similar to the air diffuser. The digesta samples (Figure 4C), however, consistently became more differentiated from the BE samples over time suggesting a stronger niche differentiation in the gut. To quantify this, we included only BE sample comparisons which were consistent in all ages – water, inlet pipe, and first feeds – and compared how the mucosal microbiomes of the fish disperse or converge toward the BE. For both gill and skin samples, the total differentiation of fish mucosal site to the three BE samples was least at 43 dph but increased with age (Figures 4D,E). The gill and skin samples were both more similar to the inlet pipe at 43 dph and became more divergent from the inlet pipe over time (137 and 430 dph). Digesta samples became more differentiated from all BE surfaces equally over time (Figure 4F). To estimate the total impact of these differences, we calculated the effect size (Figure 4G). For the gill, the dissimilarity differences across the BE samples explained 34.5% of the variation at 43 dph but then increased to 68.8% of the variation explained at 137 dph. For the skin, the largest jump in effect size occurred between 137 dph (25.6%) and 430 dph (61.5%; Figure 4G). These results indicate that niche differentiation occurs at varying rates depending on body site and that some BE microbial sources continue to have an influence on the fish mucosal microbiome throughout the lifespan of the fish, whereas other environmental sources may only be influential during early ontogeny.

### Determining Which Built Environment Surfaces Contribute to Fish Microbiome

**FIGURE 5 |:**
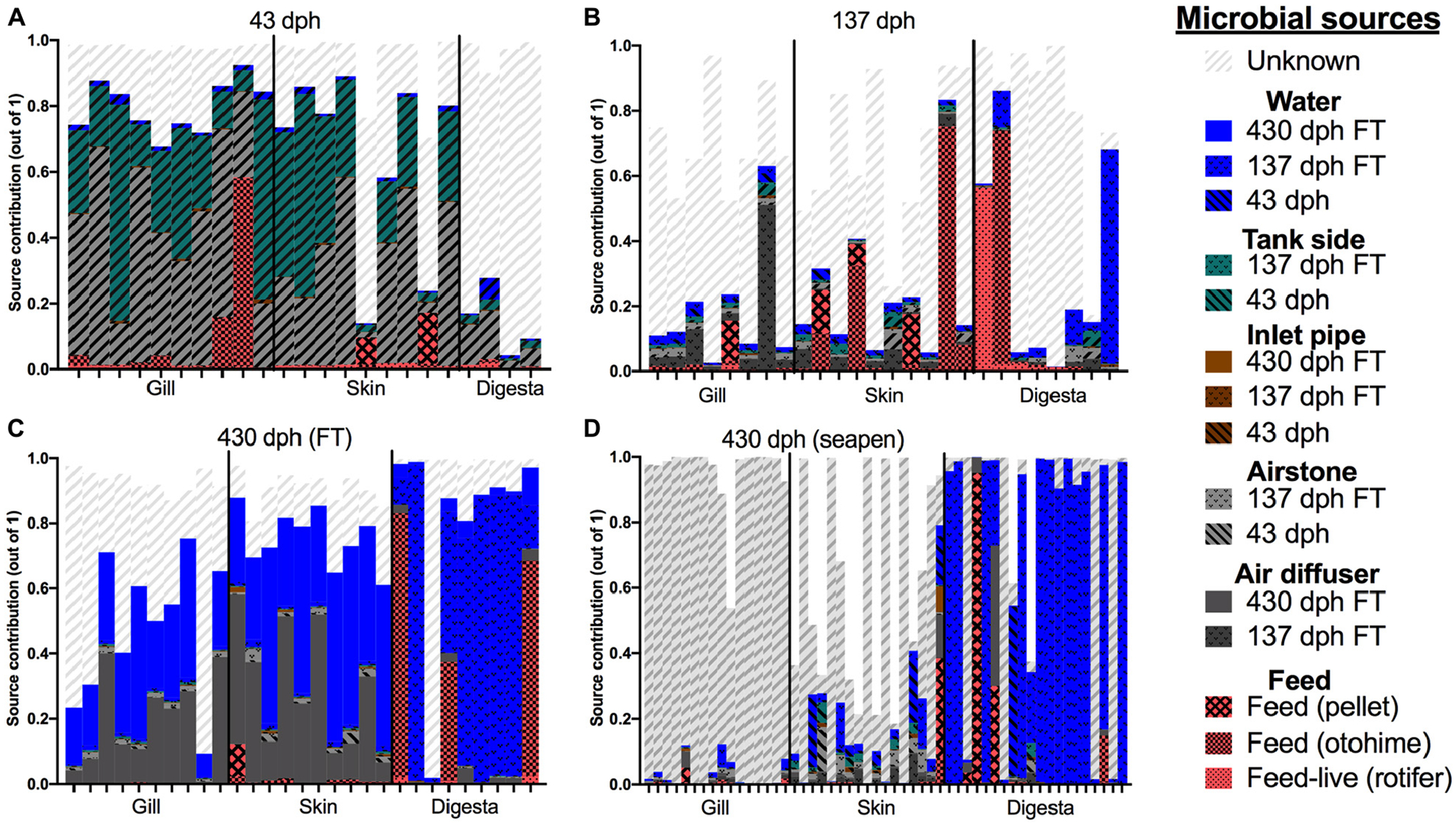
SourceTracker2 analysis of individual microbiome contributions from the built environment onto various mucosal body sites across time: **(A)** 43 dph, **(B)** 137 dph, **(C)** 430 dph indoor, and **(D)** 430 dph seapen. Features with less than 100 counts across all samples excluded. “Unknown” indicates source population was not sampled or included thus would be the percentage of a given sample which has source microbes from an unknown location or undetermined source.

To identify the extent by which the BE contributes to the mucosal microbiome of the fish, we applied the popular microbial source tracking program SourceTracker2 which uses Bayesian statistics to estimate contributions of features from various sources to sink communities. SourceTrackr2 determined that contributions of the BE varied widely depending on both the body site and the age of the fish. At 43 dph, the tank side biofilm and air stones were the biggest sources of microbes to the gill and skin of the fish larvae, while the majority of microbes in digesta samples were from unknown or unsampled sources (Figure 5A). Rotifer feeds also contributed to the gill, skin, and gut microbiomes, but to a lesser extent compared to airstone and tank side (Figure 5A). At 137 dph, gill was again influenced by the airstone and air diffusers in the BE, while higher frequencies of skin and digesta samples were colonized by microbes from feeds (Figure 5B). However, microbes from unknown sources had the largest overall contribution at 137 dph across all body sites (Figure 5B). For 430 dph fish transferred from the ocean net pen back to the land based facility, both air diffusers and the water column were the largest microbial sources to the gill and skin microbiomes (Figure 5C). For the 430 dph net pen reared fish, gill, and skin samples were primarily colonized by microbes from unknown sources followed by small proportions from air diffusers, airstones, and water from pre-transfer. Common planktonic marine microbes from sea water and netpen biofouling were not collected in this study and thus is likely a meaningful “source” which would fall into “unknown sources” in this study. Interestingly, digesta samples for both 430 dph seapen and 430 dph indoor fish were primarily colonized from water samples from the 137 dph (Figures 5C,D). This would suggest that the water community which fish are exposed to prior to transfer to ocean net pen (at around 137 dph) is very important to the gut microbiome colonization and that these microbes remain in the gut even after long term growout in seapens. The finding that the microbiome of the fish digesta originates primarily from water sources rather than feed sources is intriguing. It is important to note, however, that the feeds used in this study were normal extruded pellet feeds with no added probiotics. Results from the Sourcetracker2 analysis reinforce and support the observations from the beta diversity comparisons, that fish mucosal sites are influenced uniquely by the BE which also show succession patterns as a function of age.

### 430 dph Seapen vs. Indoor

One of the primary questions in this dataset is understanding how the surrounding environment influences mucosal microbiomes. Specifically, we were interested in understanding the specificity and stability of these microbial communities as a function of ontongeny. To compare fish of the same age (430 dph) and genetic cohort, we sampled fish which were being reared in ocean net pens along with fish which had been in seapens but were brought back to the indoor facility. Digesta samples were previously shown to have large decreases in alpha diversity at 430 dph particularly when comparing the fish in the seapen vs the indoor fish. Interestingly, much of this microbial diversity loss can be attributed to a single uncultured representative ASV, from the family Mycoplasmataceae (phylum Tenericutes, class Mollicutes), which becomes more dominant in the fish gut with age especially in the outdoor seapen. This ASV was observed in 100% of the 430 dph fish yet was found in only 75% of the 43 dph and 137 dph fish, while less frequently observed in the BE (Supplementary Figure 5a). At 430 dph this ASV made up a large proportion of total reads in the seapen (mean = 0.71) and FT indoor tank (0.60) fish but significantly less abundant in younger fish at 137 dph (0.14) and 43 dph (0.02; Supplementary Figure 5b). Thus, although the Mycoplasmataceae is present in younger fish, the proportion of reads is much smaller. Since these are proportions, it’s important to realize that this does not implicate a biomass change, but only representation in comparison to total microbial diversity.

## DISCUSSION

Seafood is an important source of protein globally which has led to the steady positive growth in aquaculture over the past 30 years. Marine finfish production has tremendous opportunity for growth (Gentry et al., 2017) yet challenges and concerns have arisen over the sustainability of such practices (Bush and Oosterveer, 2019). One of the primary concerns is animal welfare and preventing disease transmission from farmed fish to wild stocks (Bush and Oosterveer, 2019; Weitzman et al., 2019). A potential solution to antibiotic overuse in agriculture is the promotion of probiotics. The mucosal microbiome is an important component of fish health as microbes colonizing the gill, skin, and gastrointestinal tract can either be a source of infection or inversely, protect the animal from infection by inhibiting the colonization of pathogens, producing antimicrobial compounds, or eliciting an immune response (Gomez et al., 2013). Our research sought to evaluate how the mucosal microbiome develops and to estimate its stability in different body sites over time in the economically important cultured marine fish *S. lalandi*. We describe the potential sources of microbes from the “BE” (hatchery surfaces) that drive these changes across three unique body sites including the gill, skin, and digesta communities. Previous fish microbiome studies have focused primarily on one body site at a time, particularly the gut, while our approach aimed to more fully describe diversity dynamics across multiple mucosal body sites.

Gill microbiomes were the most sensitive to changes in the indoor and outdoor culture environment followed by skin with digesta demonstrating a more deterministic or enriched microbiome with ontogenic development. Specifically, while both gill and skin microbial communities increased in diversity with age, the digesta decreased. The progression of decreasing microbial diversity in the fish gut samples suggest that the gut environment is more deterministic rather than stochastic in microbial community composition. Conversely, the gill and skin generally increase in diversity with age which could be due to additive exposure and increased surface area over time. In addition to variable exposure to the external environment, individual body sites maintain unique physical and chemical properties that confer selection for specific microbial groups. Neutral (stochastic) theory ascribes that biodiversity formation and change over time occurs from random dispersal and exposure events and while it is largely conceptualized in macrofauna and flora (Hubbell, 2011), it can also be applied to microbial communities (Sloan et al., 2006). In contrast, a niche-based (deterministic) model describes how select species evolve and adapt to certain conditions as the result of interspecies interactions and niche differentiation. In this study, we demonstrate that while the gill and skin do have unique microbial communities, the processes for microbial colonization are largely stochastic whereas the gut environment demonstrates a more deterministic process for microbial colonization. In adult Atlantic salmon sampled from marine net pens, gut microbial diversity decreased as the age of fish was increased while the presence of most individual gut microbes were random and only a few deterministic, which was primarily driven by Mycoplasma (Heys et al., 2020). In zebrafish (Burns et al., 2016) and sturgeon (Abdul Razak and Scribner, 2020), both freshwater fish, higher proportions of gut microbes were non-neutral or deterministic as fish matured (older age). In catfish skin microbiomes, geographic location drove community composition with most microbes being neutral (Chiarello et al., 2019).

The implications of different body sites demonstrating a more neutral or deterministic microbiome is important for understanding both the impact of environmental change on wild fish stocks as well as improving aquaculture production. Negative anthropogenic impacts to the marine environment include contaminant and nutrient pollution which can cause disturbances of primary productivity. In a wild marine fish, the Pacific chub mackerel, the composition of external mucosal microbiomes of gill and skin were most influenced by temporal changes, coinciding with temperature, along with gill alpha diversity positively correlated with age (Minich et al., 2020a). The gill is an important organ for excretion of nitrogenous waste (Sayer and Davenport, 1987; Wilkie, 2002) and gas exchange which is critical for highly active swimming fish like *Seriola* spp. (Yamamoto et al., 1981; Roberts and Rowell, 1988). In aquaculture settings, microbes which produce compounds causing off-flavor in flesh (Auffret et al., 2013) have been found to be enriched and primarily taken up through the gills of fish (From and Hørlyck, 1984; Klausen et al., 2005). Since the gill is a critical component of maintaining homeostasis, and in this study appears most susceptible to changing environmental conditions, further research is needed to understand how changes in the microbiome may negatively or positively impact fish physiology. Additionally, skin is an important physical barrier for disease prevention. The skin microbiomes of two coastal pelagic marine fish, *Scomber japonicus* and *S. lalandi*, were strongly influenced by increased temperature that coincided with increased proportions of a potential marine pathogen, *Photobacterium* spp. (Horlick et al., 2020; Minich et al., 2020a).

Body site microbiomes of *S. lalandi* were most similar to the BE surfaces at the earliest age (43 dph). As fish aged, digesta samples diverged from all BE surfaces, while gill and skin were differentially influenced by specific BE surfaces. In Atlantic salmon reared in freshwater indoor hatcheries, microbial diversity from both the tank side and water column were highly correlated with the fish skin and gut, but not other BE surfaces (Minich et al., 2020b). Understanding which surfaces likely contributed to the various body sites over time was calculated using SourceTracker2 analysis. At 43 dph, the biofilm from the tank side along with the aeration equipment (airstones) were the largest contributors to the gill and skin communities whereas much of the digesta microbes were from unknown sources. Aeration equipment in tilapia culture has been implicated as a source of *Acinetobacter* in culture systems (Grande Burgos et al., 2018). While feed had a marginal impact on the microbial community of the various fish body sites, it was not consistent and was generally lower than the surrounding BE surfaces. Although diet has been shown to have a strong influence on gut microbiome development (Nayak, 2010; Tarnecki et al., 2017), the importance of live feeds as contributors to the gut microbiome is debated (Ringø, 1999; Bakke et al., 2013). One explanation is that the microbes colonizing the live feeds have low specificity for successful colonization of the fish gut. Likewise, since the overall exposure to and density of BE surfaces and associated microbes, including the water, is much greater than that of the live feeds (Walburn et al., 2019), feed-associated taxa may be outcompeted in the gut environment.

A unique opportunity of this study was to compare mature fish (430 dph) from an ocean net pen to fish that had been in the ocean but were transported back to an indoor system to be used as broodstock. We are not aware of any other study which has looked at the microbiome transition from ocean to indoor in a marine fish. Selective breeding programs rely on the ability to develop broodstock which are used to maintain genetic lines from previous grow out populations (Symonds et al., 2014). Ocean net pen fish generally had lower microbial diversity than indoor reared fish for all fish body sites, but was most pronounced in the gill. This further suggests that the mucosal, even in adult fish that are least susceptible to BE impacts, has a high capacity to change which is critical when considering time scales for probiotic effects (Vadstein et al., 2018; Dawood et al., 2019; Ramírez et al., 2019). Probiotic treatments in fish are common but little is known about dosage for a given treatment along with frequency of administration for having a lasting effect. If the normal microbial community of a fish gill or skin can change rapidly, this would suggest that a sustained administration rather than a “one-time treatment” would be required for maintaining mucosal health in fish. For gill and skin communities, the water column and aeration surfaces contributed the most for indoor reared fish while fish reared in the net pens had many bacteria of unknown sources, presumably from the ocean, e.g., seawater. Digesta samples, however, were primarily colonized by hatchery water associated microbiota and to a lesser extent feeds. The opposite explanation is also true that in land-based systems, fish feces could be contributing more to the water column microbiome as compared to the oceanic conditions where feces is more quickly exported out of the system. These vast differences and the speed at which microbiomes develop and change is a plausible explanation for differences between wild and farmed *Seriola* (Ramírez and Romero, 2017). The most abundant microbe in the *Seriola* digesta was an unresolved Mycoplasmataceae which was strongly associated with transfer of fish from indoor rearing systems to the ocean net pen. Mycoplasma are important gut microbes which can colonize the gut very early in development. Several plausible explanations exist for this observation. First, it is possible that in land-based systems, fish are simply not as heavily exposed to Mycoplasma. Second, it is possible that Mycoplasma microbial density or diversity is higher in ocean net pen systems compared to the indoor system thus allowing the Mycoplasma to dominate the gut microbiome. Lastly, an alternative explanation is that Mycoplasma outcompetes other microbes in the fish gut especially as the fish increase in age. However, since the data are compositional, it is not possible to determine absolute microbial densities thus requiring additional experimentation to resolve. All mucosal environments were influenced by the BE over time with the strongest effects at early fish development. Digesta samples in particular became less influenced by the BE over time and demonstrated a strong selective or deterministic pressure on microbiome development with increasing age. This progression of decreasing microbial diversity in the fish gut suggests that the gut environment is more deterministic rather than stochastic in microbial community composition whereas the gill and skin generally increase in diversity with age which could be due to additive exposure.

One of the limitations of this study is that we did not perform quantitative measures of the microbial communities. Part of the reason for this is that these methods can often involve invasive or destructive sampling of tissues. Since we largely utilize non-invasive sampling techniques, at least for the fish samples, performing quantitative measures is a challenge. Nonetheless, future studies should focus on developing non-invasive methods for accessing the quantitative measures of microbial quantities in both the BE and the fish mucous.

## Supplementary Material

Supplementary Material

## Figures and Tables

**TABLE 1 |:** Multivariate statistical comparison of impacts of rearing system across gill and skin (PERMANOVA, 999 permutations).

Unweighted Unifrac		YTK_tank_system		
Body_site		*n*	*P*	*F*
Gill	FT vs RAS BioGill vs RAS MBBR	35	0.001	2.72
	FT vs RAS BioGill	23	0.001	2.82
	FT vs RAS MBBR	24	0.001	3.29
	RAS BioGill vs RAS MBBR	23	0.001	1.95
Skin	FT vs RAS BioGill vs RAS MBBR	32	0.002	1.73
	FT vs RAS BioGill	20	0.565	0.94
	FT vs RAS MBBR	21	0.001	2.21
	RAS BioGill vs RAS MBBR	23	0.002	2.08
Weighted normalized Unifrac		YTK_tank_system		
Body_site		*n*	*P*	*F*
Gill	FT vs RAS BioGill vs RAS MBBR	35	0.001	11.01
	FT vs RAS BioGill	20	0.001	17.43
	FT vs RAS MBBR	21	0.001	11.55
	RAS BioGill vs RAS MBBR	23	0.018	3.18
Skin	FT vs RAS BioGill vs RAS MBBR	32	0.182	1.60
	FT vs RAS BioGill	20	0.256	1.62
	FT vs RAS MBBR	21	0.038	2.87
	RAS BioGill vs RAS MBBR	23	0.413	0.83

## Data Availability

The datasets presented in this study can be found in online repositories. The names of the repository/repositories and accession number(s) can be found below: Data is publicly available on European Nucleotide Archive (EBI: ERP120036) and through Qiita (Study ID: 12227 and Analysis ID 25157).
