# Supplementary material for: Impacts of the Marine Hatchery Built Environment, Water and Feed on Mucosal Microbiome Colonization Across Ontogeny in Yellowtail Kingfish, Seriola lalandi
Source: Front Mar Sci. Author manuscript; Available in PMC 2022 Oct 14. (PMC9563383; doi:10.3389/fmars.2021.676731)
Supplement: Supplementary Material [file NIHMS1806685-supplement-Supplementary_Material.pdf]

Supplementary Material

Supplemental Table S1. Sample success across sample_types

experiment	age	tank	sample_type	N	>=5000 reads<br>N_success
1	130	FT	gill	12	12
1	130		skin	12	9
1	130		tank water	1	1
1	130		tank side	1	1
1	130		inlet pipe	1	1
1	130		air diffuser	1	1
1	130		airstone	1	1
1	130	RAS BioGill	gill	12	11
1	130		skin	12	11
1	130		inlet water	1	1
1	130		tank water	1	0
1	130		tank side	1	1
1	130		inlet pipe	1	1
1	130		air diffuser	1	1
1	130	RAS MBBR	gill	12	12

1	130		skin	12	12
1	130		inlet water	1	1
1	130		tank water	1	0
1	130		tank side	1	1
1	130		inlet pipe	1	1
1	130		air diffuser	1	1
2	43	WL3 &WL6	gill	12	10
2	43		skin	12	9
2	43		digesta	12	4
2	43		tank water	2	2
2	43		tank side	2	1
2	43		inlet pipe	2	1
2	43		airstone	2	1
2	137	FT	gill	12	8
2	137		skin	12	10
2	137		digesta	12	8
2	137		tank water	1	1
2	137		tank side	1	1
2	137		inlet pipe	1	1
2	137		air diffuser	1	1

2	137		airstone	1	1
2	430	seapen FT	gill	12	10
2	430		skin	12	10
2	430		digesta	12	9
2	430		tank water	1	1
2	430		tank side	1	0
2	430		inlet pipe	1	1
2	430		air diffuser	1	1
2	430	seapen	gill	20	16
2	430		skin	20	17
2	430		digesta	20	20
2		feed	artemia 500ul	1	0
2			unenriched rotifer 500ul	1	1
2			enriched rotifer espresso 500ul	1	1
2			Otohime b1	1	1
2			Otohime b2	1	1
2			Otohime C1	1	1
2			Otohime c2	1	1
2			0.5 mm Nutra feed	1	1
2			0.8 mm biomar feed	1	1

2		Ridley 1.5mm	1	1
2		Ridley 3 mm	1	1
2		Ridley 3 mm w vit c	1	1
2		Skretting 9mm	1	1
	NA	Negative controls	7	3
	NA	Positive controls	12	7
	Total		304	246

Table S2. Multivariate statistical comparison of microbial communities by a) impacts of age for each independent body site and b) differences across body sites for each unique age group (PERMANOVA, 999 permutations)

a	UniFrac	Filtered_group	test_group	n	P	F
	unw	43 dph	sample_type	29	0.001	4.62
	unw	137 dph	sample_type	26	0.023	1.29
	unw	430 dph tank	sample_type	29	0.001	4.62
	unw	430 dph sea	sample_type	53	0.001	6.67
	w	43 dph	sample_type	29	0.001	5.57
	w	137 dph	sample_type	26	0.003	3.11
	w	430 dph tank	sample_type	29	0.001	18.39
	w	430 dph sea	sample_type	53	0.001	32.29

b	unw	Gill	ylk_tank_system	44	0.001	5.90
	unw	Skin	ylk_tank_system	46	0.001	3.51
	unw	Digesta	ylk_tank_system	41	0.001	3.47
	w	Gill	ylk_tank_system	44	0.001	14.53
	w	Skin	ylk_tank_system	46	0.001	7.72
	w	Digesta	ylk_tank_system	41	0.001	6.78

UniFrac: unweighted unifrac = 'unw', weighed normalized unifrac = 'w'

ylk_tank_system: 43 dph, 137 dph, 430 dph 'tank', 430 dph 'seapen'

sample_type: fish gill, fish skin, fish gut

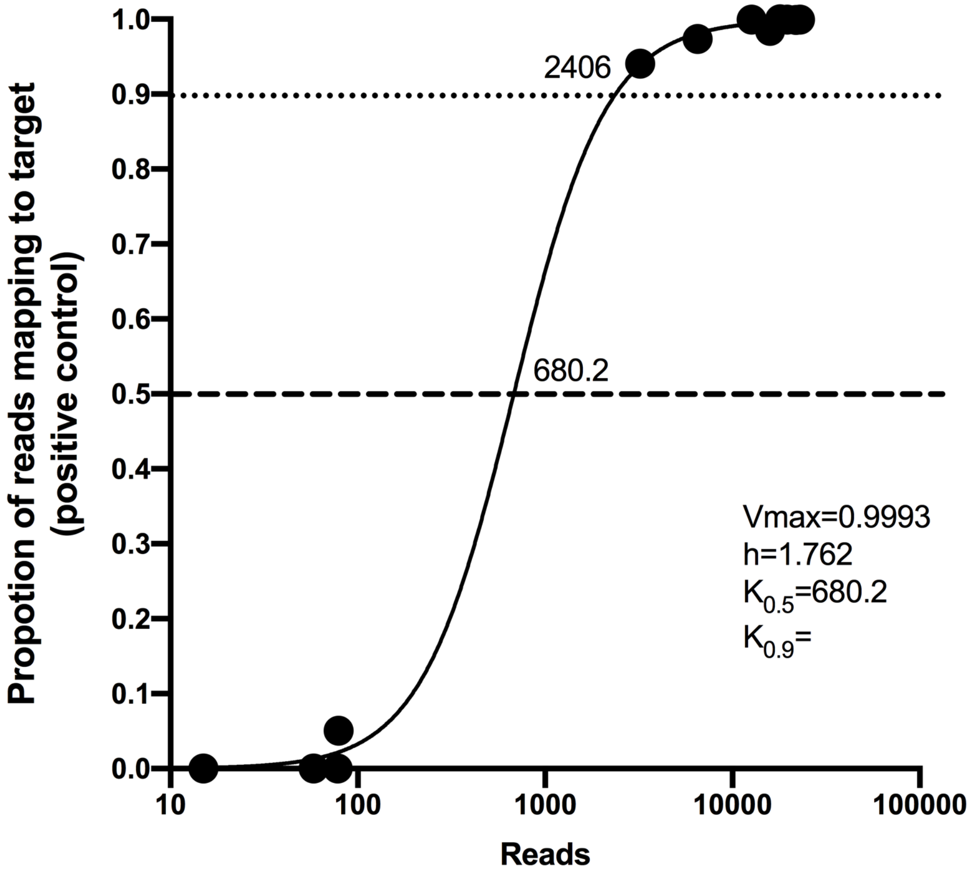

Supplementary Figure 1. Sample exclusion cutoff determination using Katharoseq method for limit of detection.

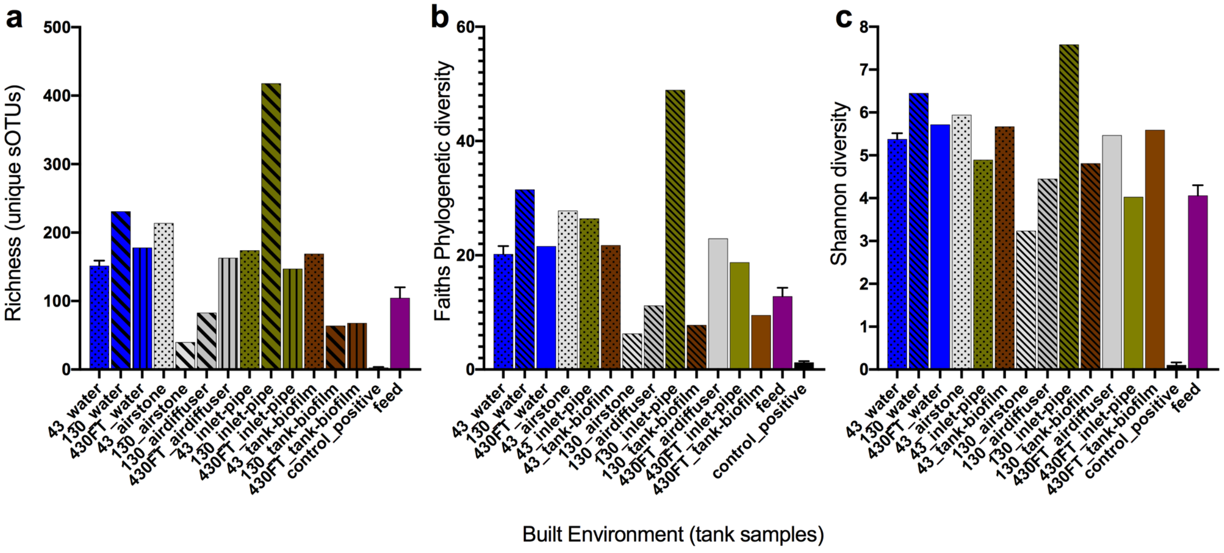

Supplementary Figure 2. Alpha diversity measures a) richness, b) Faith's Phylogenetic diversity, and c) Shannon across hatchery surfaces

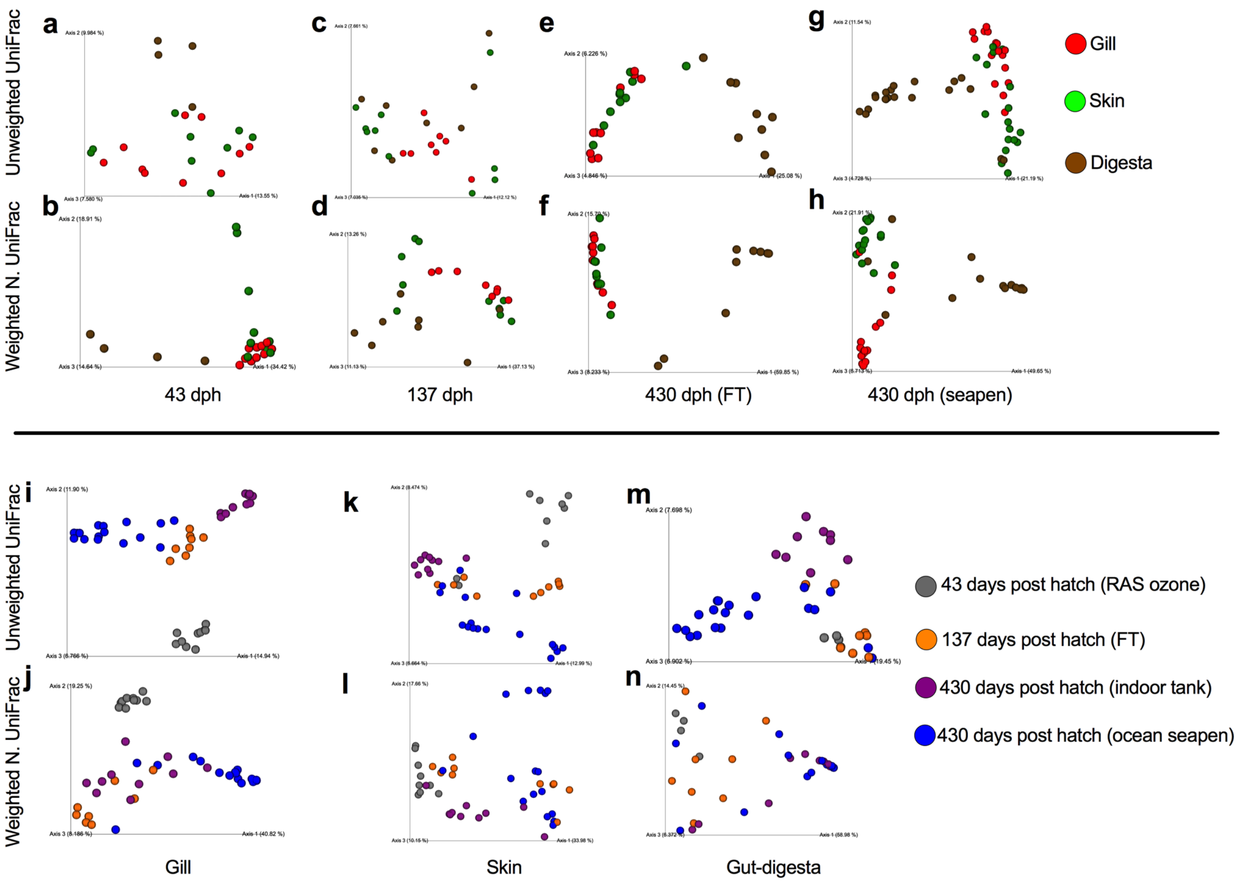

Supplementary Figure 3. Beta diversity visualizations using PCoA plots of both Unweighted and Weighted Normalized Unifrac distances. Comparisons of microbial diversity across body sites using Unweighted and Weighted Unifrac distances (respectfully) independent for each age group: a) b) 43 dph; c) d) 137 dph; e) f) 430 dph indoor tank; g) h) 430 dph seapen. Comparisons of microbial diversity across rearing conditions or age using Unweighed and Weighted Unifrac distances grouped independently for body sites: i) j) gill; k) l) skin; m) n) digesta.

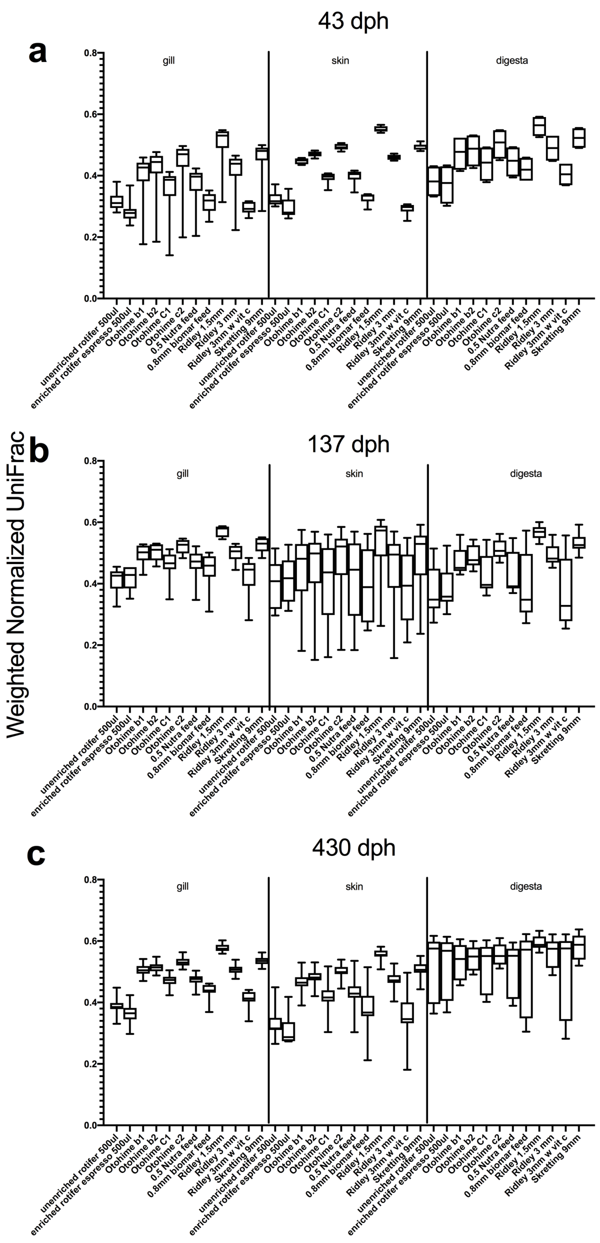

Supplementary Figure 4. Beta diversity comparisons (weighted normalized UniFrac) of fish mucosal sites (gill, skin, and digesta) to 12 different feeds used throughout the production process (order left to right from earliest feed to late feed). Each comparison conducted per age of fish: a) 43 dph, b) 137 dph, and c) 430 dph.

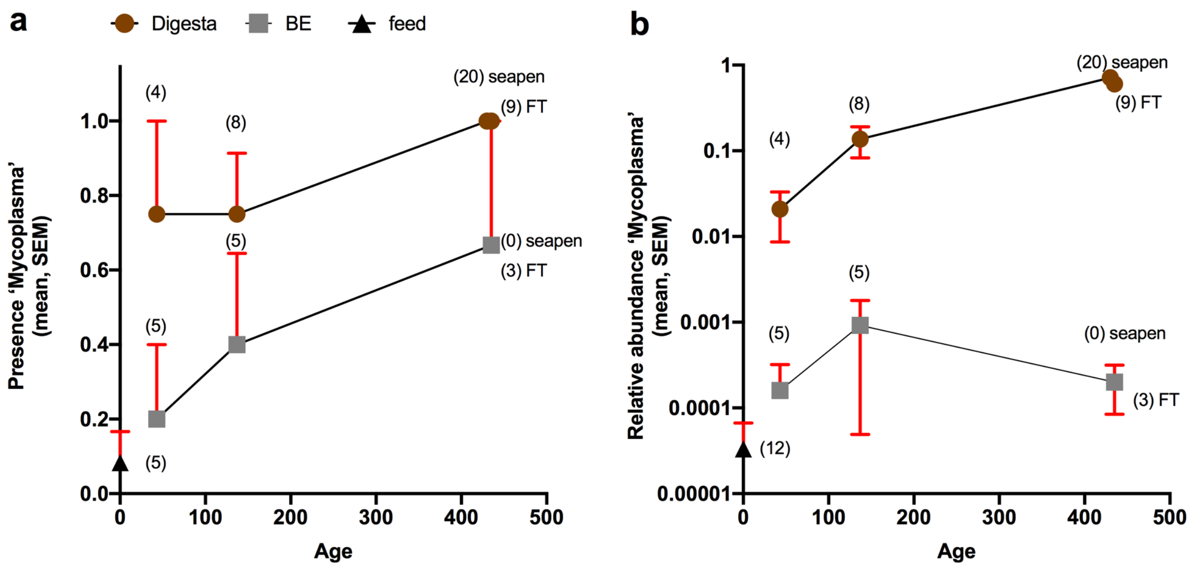

Supplementary Figure 5. Distribution of highly abundant 'unannotated' Mycoplasmataceae in digesta and built environment samples across fish age. A) Presence and B) relative abundance distribution of Mycoplasmataceae in fish and environment samples.
